# Omega-3 Polyunsaturated Fatty Acids Protect Neural Progenitor Cells against Oxidative Injury

**DOI:** 10.3390/md12052341

**Published:** 2014-04-29

**Authors:** Qiang Liu, Di Wu, Na Ni, Huixia Ren, Chuanming Luo, Chengwei He, Jing-Xuan Kang, Jian-Bo Wan, Huanxing Su

**Affiliations:** 1 State Key Laboratory of Quality Research in Chinese Medicine, Institute of Chinese Medical Sciences, University of Macau, Macao 999078, China; E-Mails: yb37525@umac.mo (Q.L.); mb35801@umac.mo (D.W.); mb15844@umac.mo (N.N.); yb27526@umac.mo (H.R.); 15989166366@163.com (C.L.); chengweihe@umac.mo (C.H.); jbwan@umac.mo (J.-B.W.); 2 Laboratory for Lipid Medicine and Technology, Massachusetts General Hospital and Harvard Medical School, Boston, MA 02114, USA; E-Mail: jxkang@mgh.harvard.edu (J.-X.K.)

**Keywords:** oxidative stress, DHA, fat-1, neural progenitor cells

## Abstract

The omega-3 polyunsaturated fatty acids (ω-3 PUFAs), eicosapentaenoic acid (EPA) and docosahexaenoic acid (DHA), derived mainly from fish oil, play important roles in brain development and neuroplasticity. Here, we reported that application of ω-3 PUFAs significantly protected mouse neural progenitor cells (NPCs) against H_2_O_2_-induced oxidative injury. We also isolated NPCs from transgenic mice expressing the *Caenorhabditis elegans fat-1* gene. The *fat-1* gene, which is absent in mammals, can add a double bond into an unsaturated fatty acid hydrocarbon chain and convert ω-6 to ω-3 fatty acids. Terminal deoxynucleotidyl transferase dUTP nick end labeling (TUNEL) staining showed that a marked decrease in apoptotic cells was found in *fat-1* NPCs after oxidative injury with H_2_O_2_ as compared with wild-type NPCs. Quantitative RT-PCR and Western blot analysis demonstrated a much higher expression of nuclear factor erythroid 2-related factor 2 (Nrf2), a master transcriptional factor for antioxidant genes, in *fat-1* NPCs. The results of the study provide evidence that ω-3 PUFAs resist oxidative injury to NPCs.

## 1. Introduction

Oxidative stress-induced neuronal apoptosis plays a critical role in the pathogenesis of stroke and neurodegenerative diseases [1,2]. Oxidants, such as hydrogen peroxide and free radicals, produce cell damage by inducing production of reactive oxygen species (ROS) and activate an inflammatory response [3]. Several studies confirmed the transcription factor nuclear factor erythroid 2-related factor 2 (Nrf2) represents an important cellular protective mechanism against oxidative stress over the Nrf2-ARE pathway [4,5]. Activation of Nrf2 signaling induces the transcriptional regulation of ARE-dependent expression of various antioxidant and phase II detoxification enzymes, which include hemeoxygenase-1 (HO-1), NAD(P)H quinine oxidoreductase 1 (NQO-1), glutamate-cysteine ligase modifier subunit (GCLM), and glutamate-cysteine ligase catalytic subunit (GCLC) [6].

The long-chain omega-3 polyunsaturated fatty acids (ω-3 PUFAs) from fish oil, for example, Docosahexaenoic acid (DHA), are highly enriched in the brain and play a key role in brain development and repair under many conditions [7,8]. Dietary DHA has been suggested to improve neuronal development [9], restore and enhance cognitive functions [10,11,12], and protect against beta-amyloid production, accumulation, and potential downstream toxicity in an aged Alzheimer mouse model [13]. DHA have also been shown to exert a beneficial effect on ROS related cellular damage [14]. These studies indicate that omega-3, such as DHA, can increase neural resistance to various types of insults.

Mammals are unable to synthesize ω-3 PUFAs de novo and must rely on a dietary source of these essential fatty acids. The *C. elegans fat-1* gene encodes an *n*-3 fatty acid desaturase that converts ω-6 to ω-3 PUFA, which could significantly reduce the omega-6/omega-3 fatty acid ratio [15]. The *fat-1* transgenic mice are rich in endogenous ω-3 PUFAs, specifically in the brain, with a reduction in ω-6 fatty acids, which provides an optimal model to evaluate the actions of ω-3 PUFAs [16,17].

In the present study, we investigated whether ω-3 PUFAs could protect neural progenitor cells (NPCs) against oxidative injury. NPCs are multipotent with a broad self-renewing potential and with the capacity to generate neurons, astrocytes and oligodendrocytes. Their inherent biological properties of NPCs provide multiple potentials to treat various neurological dysfunctions. Our results provide evidence that both exogenous and endogenous ω-3 PUFAs can resist oxidative injury to NPCs.

## 2. Results

### 2.1. *In Vitro* Characterization of NPCs^WT^ and NPCs^fat-1^

**Figure 1:**
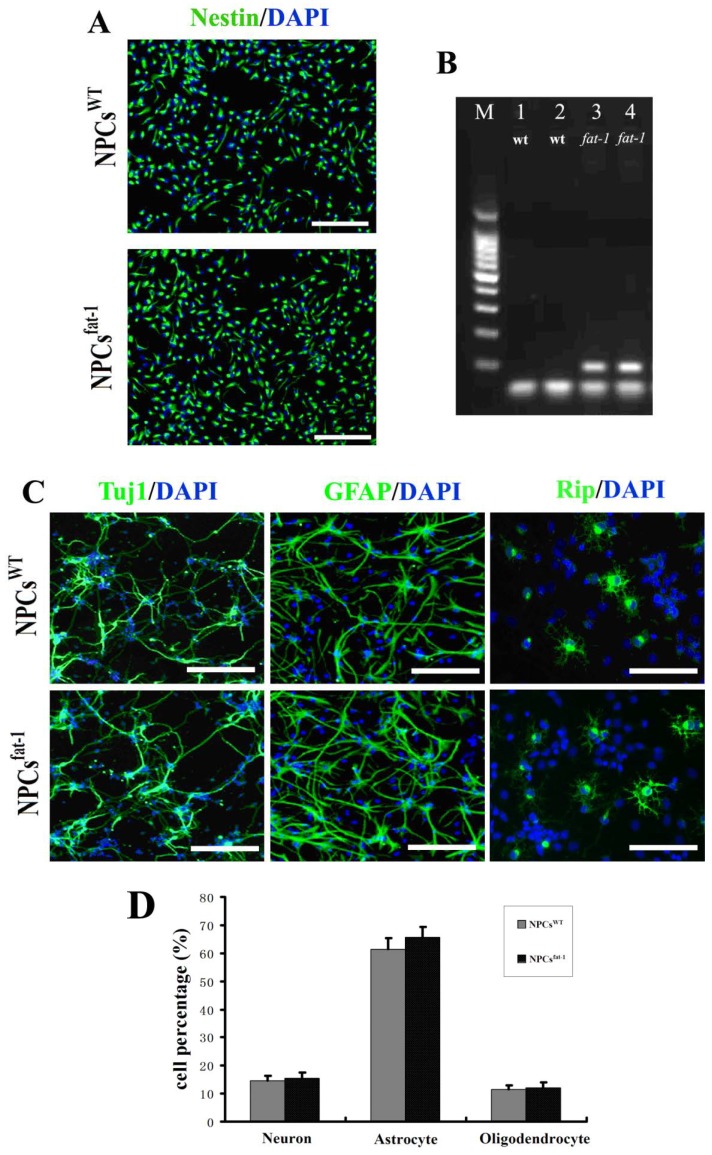
Characterization on NPCs^WT^ and NPCs^fat-1^. (**A**) The purity of neural progenitor cells (NPCs) was identified by Nestin staining and nuclei were counter-stained with DAPI. More than 95% of NPCs^WT^ or NPCs^fat-1^ were nestin-positive cells; (**B**) Gel electrophoresis of PCR products using primers for *fat-1* gene. Wild-type controls (lanes 1 and 2) and positive *fat-1* specimens (lanes 3 and 4); (**C**) Both NPCs^WT^ and NPCs^fat-1^ were shown to successfully differentiate into Tuj1-positive neurons, GFAP-positive astrocytes, and Rip-positive oligodendrocytes; (**D**) The bar graph showing the percentage of neural cells differentiated from NPCs at the 5th day in the differentiating medium. Scale bar: 250 μm in (**A**) and 100 μm in (**C**).

Neural progenitor cells (NPCs) are self-renewing, multipotent cells that could be effectively differentiated into neurons, astrocytes, and oligodendrocytes [18]. With bFGF-supplemented culture medium, both NPCs^WT^ and NPCs^fat-1^ cells showed bipolar or multipolar morphology with small cell bodies. Nestin is an intermediate filament protein and widely used as a specific marker for NPCs. Immunostaining showed that more than 95% cells in both NPCs^WT^ and NPCs^fat-1^ culture were nestin-positive, confirming that the majority of NPCs^WT^ and NPCs^fat-1^ were immature (Figure 1A). PCR analysis demonstrated the high expression of *fat-1* in NPCs^fat-1^ (lanes 3 and 4) while no expression in NPCs^WT^ (lanes 1 and 2) (Figure 1B). To study differentiation potential of NPCs^WT^ and NPCs^fat-1^ *in vitro*, bFGF was replaced with 1% FBS in the cell culture medium and NPCs began to differentiate. At the 5th day, cultures with this differentiating medium, both NPCs^WT^ and NPCs^fat-1^ were successfully differentiated into Tuj1-positive neurons, GFAP-positive astrocytes and Rip-positive oligodendrocytes with similar differentiation capacities (NPCs^WT^: 14.7% neurons, 61.3% astrocytes, and 11.5% oligodendrocytes; NPCs^fat-1^: 15.4% neurons, 65.6% astrocytes, and 12.1% oligodendrocytes) (Figure 1C,D).

### 2.2. DHA Protected NPCs against H_2_O_2_-Mediated Apoptosis

**Figure 2:**
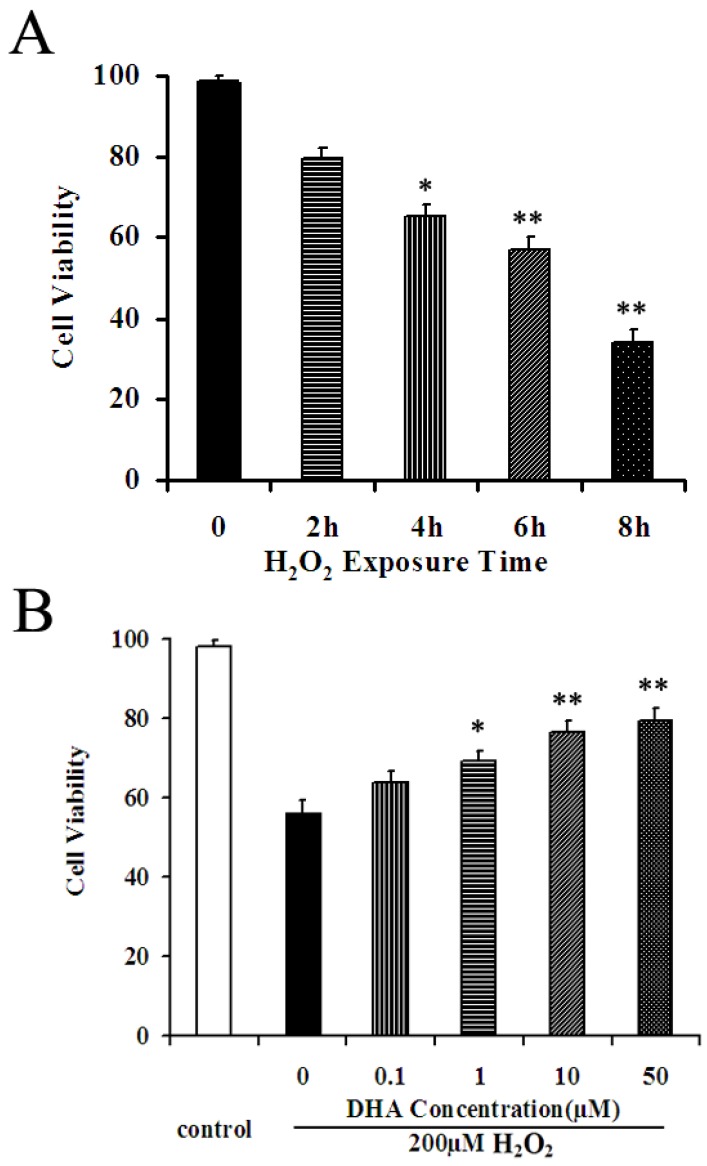
DHA pretreatment reduced oxidative stress on cultured NPCs. (**A**) WST-8 assays revealed that incubation of 200 μM H_2_O_2 _has caused a significant cytotoxicity in a time-dependent manner. (**B**) DHA prevented H_2_O_2_-induced cell death in a concentration-dependent manner. Cell viability was presented as a percentage of control, and each value represents the mean ± SD of three independent experiments; * *p* < 0.05 and ** *p* < 0.001 *versus* control.

To evaluate the effect of H_2_O_2_ on cell viability, we first incubated NPCs with 200 μM H_2_O_2_ and investigated cell viability at different time points. Cell viability was measured using the WST-8 assay. A 200 μM concentration of H_2_O_2_ in culture was used to establish the oxidative injury model according to a previous study reporting that cultured NPCs exposed to H_2_O_2_ at this concentration was sufficiently induced to undergo apoptosis [19]. As shown in Figure 2A, cell viability was significantly reduced in a time-dependent manner. Incubation of 200 μM H_2_O_2_ for 6 h caused an approximate 50% cell loss, which was considered to be an optimal oxidative injury model for investigating drug effects.

We then investigated the neuroprotective effects of exogenous DHA on H_2_O_2_-mediated apoptosis. NPCs^WT^ at the confluence of 80% was pretreated with DHA (0, 0.1, 1, 10, and 50 μM) for 2 h and then suffered an oxidative injury induced by incubation of 200 μM H_2_O_2_ for 6 h. WST-8 assay revealed that the cell viability increased in a concentration-dependent manner: The pretreatment of 1 μM DHA increased the cell viability by 22.1% as compared to vehicle control (*p* < 0.05, Figure 2B), while the pretreatment of 10 μM and 50 μM increased the cell viability by 35.6% and 36.2%, respectively, as compared to the vehicle control (*p* < 0.001, Figure 2B).

### 2.3. NPCs^fat-1^ Prevented H_2_O_2_-Mediated Apoptosis

**Figure 3:**
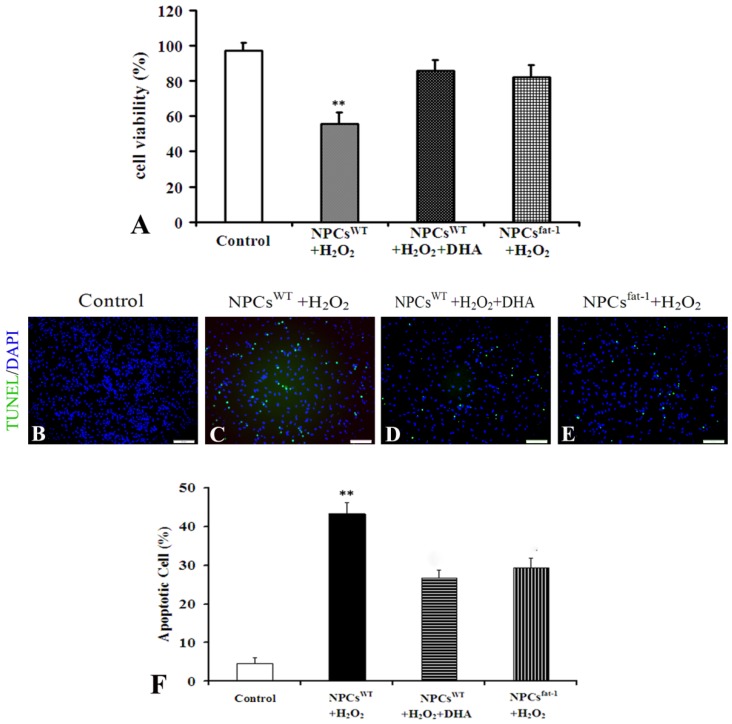
NPCs^fat-1^ attenuated H_2_O_2_-mediated apoptosis. (**A**) The cell viability of NPCs was assessed after exposure to H_2_O_2_ for 6 h by WST-8 analysis. Each value represents the mean ± SD of three independent experiments (*n* = 3, ** *p* < 0.01 *versus* other groups); (**B**–**E**) Representative photomicrographs of TUNEL assay; (**F**) Quantitative analysis was carried out by measuring TUNEL-positive cells in each group. Figures were selected as representative data from three independent experiments. Cell apoptosis was significantly reduced in DHA-pretreated NPCs^WT^ and NPCs^fat-1^. Each value represents the mean ± SD of three independent experiments (*n* = 3, ** *p* < 0.01 *versus* other groups). Scale bar: 75 μm.

We further investigated anti-oxidative effects of endogenous ω-3 PUFAs in NPCs. NPCs^fat-1^ were isolated from *fat-1* mice, which are rich in endogenous ω-3 PUFAs, specifically in the brain [16,17]. WST-8 assay showed that NPCs^fat-1^ exhibited a potent anti-oxidative effect similar to that found in the DHA-treated NPCs^WT^ group when exposed to H_2_O_2_ for 6 h. The cell viability of these two groups was significantly increased as compared to the vehicle control (Figure 3A). Terminal deoxynucleotidyl transferase-mediated UTP end-labeling (TUNEL) staining was also performed to detect H_2_O_2_-mediated apoptosis. Only a very small proportion of intrinsic apoptosis were detected in cultured NPCs^WT^ (Figure 3B,F). Incubation with 200 μM H_2_O_2_ for 6 h induced approximately 40% NPCs^WT^ to undergo apoptosis (Figure 3C,F), while pretreatment of NPCs^WT^ with 10 μM DHA significantly attenuated H_2_O_2_-mediated apoptosis to less than 30% (Figure 3D,F). NPCs^fat-1^ exhibited potent anti-oxidative properties, as shown by a significant decrease in apoptosis compared to NPCs^WT^ when exposed to H_2_O_2_ for 6 h (Figure 3E,F). These findings indicated that both exogenous and endogenous ω-3 PUFAs could protect NPCs against H_2_O_2_-mediated oxidative injury.

### 2.4. Expression Analyses of Nrf2-ARE Pathway Genes

**Figure 4:**
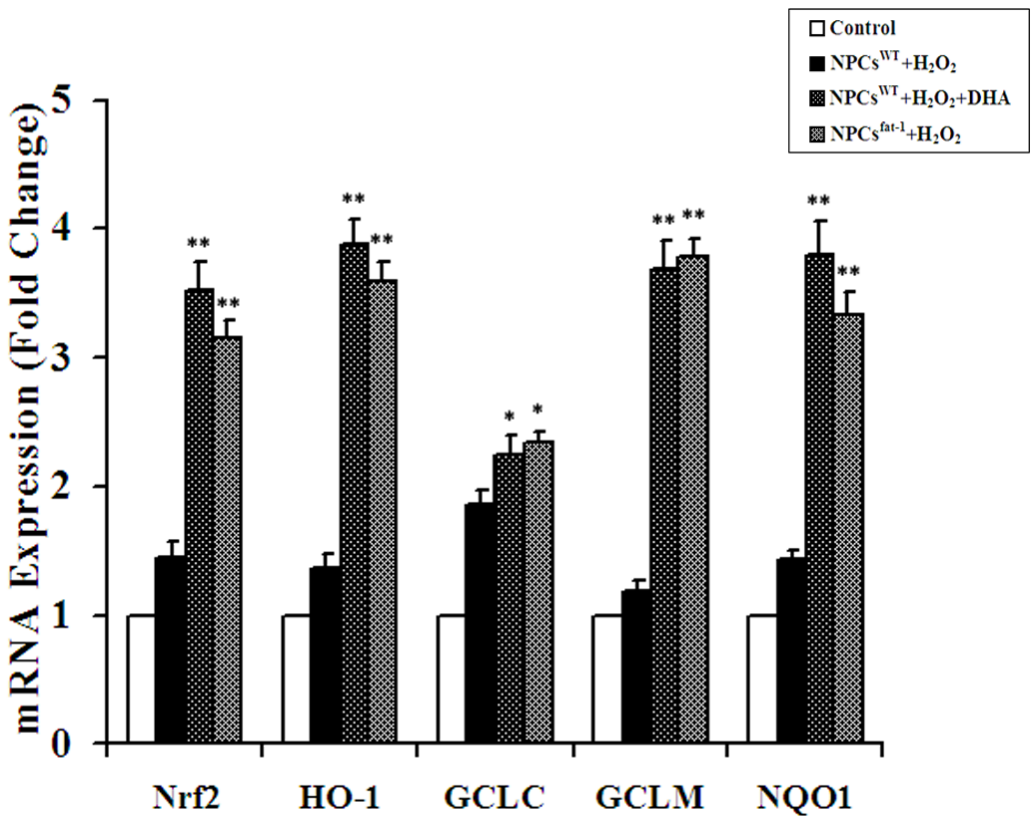
Expression analyses of Nrf2-ARE pathway genes in DHA-pretreated NPCs^WT^ and NPCs^fat-1^. Real-time RT-PCR assays showed that both DHA pretreatment and NPCs^fat-1^ induced significant increases in the transcript level of Nrf2 and its downstream gene and phase II detoxification gene transcripts HO-1, NQO-1, GCLC, GCLM when compared with the controls. Data are shown as mean ± SD (*n* = 3); * *p* < 0.05 *versus* control; ** *p* < 0.01 *versus* control.

To study the anti-oxidative mechanisms of ω-3 PUFAs against H_2_O_2_-induced apoptosis in NPCs, we first investigated whether Nrf2, the principal transcription factor that regulates the basal and inducible expression of a battery of antioxidant genes, was up-regulated after pretreatment with DHA and in NPCs^fat-1^. Real-time RT-PCR assays showed that both DHA pretreatment and NPCs^fat-1^ induced a nearly 2.5-fold increase in the transcript level of Nrf2 when compared with the controls (Figure 4). Furthermore, significant increases in the expression level of the downstream gene and phase II detoxification gene transcripts (HO-1, NQO-1, GCLC, GCLM) were found in NPCs^fat-1^ and DHA-pretreated NPCs^WT^ (Figure 4).

### 2.5. Expression Profiles of Nuclear and Cytosolic Nrf2 by Western Blot Analysis

**Figure 5:**
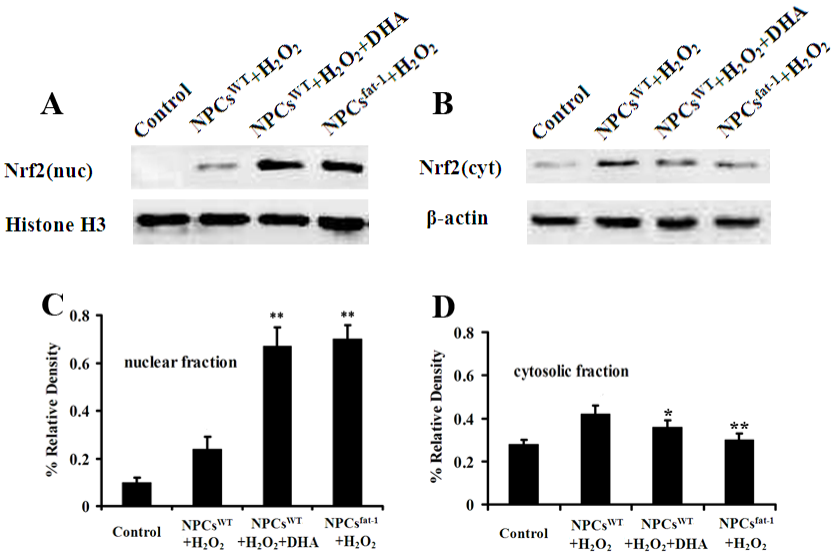
DHA pretreatment and NPCs^fat-1^ induced a significant increase in nuclear Nrf2 expression and a significant decrease in cytosolic Nrf2 expression. DHA-pretreated NPCs^WT^ and NPCs^fat-1^ were treated with 200 μM H_2_O_2_ for the indicated time points. Cells were lysed and fractionated to isolate nuclear and cytosolic fractions as indicated. Fractions were confirmed using Western blot with histone H3 for nuclear fractions (**A**) and β-actin as a marker for cytosolic fractions (**B**); Densitometry analysis showed that DHA pretreatment and NPCs^fat-1^ induced a significant increase in nuclear Nrf2 expression (**C**) and a significant decrease in cytosolic Nrf2 expression (**D**). Data are shown as mean ± SD (*n* = 3); * *p* < 0.05 *versus* control; ** *p* < 0.01 *versus* control.

Real-time RT-PCR assays demonstrated that both DHA pretreatment and NPCs^fat-1^ induced significant increases in the transcript level of Nrf2. As nuclear translocation of protein Nrf-2 is very critical in inducing gene expression of anti-oxidant genes, we then investigated the expression profiles of cytosolic and nuclear fraction Nrf2 by Western blot analysis. Consistent with real-time RT-PCR results, Western blot analysis demonstrated that both DHA pretreatment and NPCs^fat-1^ significantly increased the protein expression of nuclear Nrf2 when compared with the controls (Figure 5A,C). However, the expression of cytosolic Nrf2 in DHA pretreatment and NPCs^fat-1^ was significantly decreased when compared with the control (Figure 5B,D). These results demonstrated an obvious translocation of Nrf2 from the cytoplasm to the nucleus in DHA pretreatment and NPCs^fat-1^.

## 3. Discussion

Neurodegenerative diseases are characterized by the progressive loss of neurons and usually influence the cognitive function, movement control or muscle strength [1,2]. Neurodegenerative diseases are commonly late-onset disorders, including Alzheimer’s disease (AD), Parkinson’s disease (PD), Huntington’s disease (HD), and Amyotrophic lateral sclerosis (ALS). Oxidative stress has a significant role in the pathogenesis and/or progression of several neurodegenerative diseases and aging diseases, which are closely associated with disease-specific proteins aggregation, inflammation, mitochondrial dysfunction, and neurotoxicity [1,2,18]. Effective antioxidants have promising potential for therapeutic application. A prospective strategy in disease control has been focused on development of antioxidants as preventive and therapeutic medicine.

NPCs are largely undifferentiated cells originating in the central nervous system. They have the potential to give rise to offspring cells and efficiently differentiate into neurons, astrocytes and oligodendrocytes [19,20]. The inherent biological properties of NPCs may provide multiple strategies to treat CNS dysfunction and enable them to be an optimal model to screen antioxidants, which have therapeutic potentials for the treatment of neurological diseases.

DHA is an *n*-3 long chain PUFA, highly enriched in the central nervous system, and is critical for brain development and function. DHA is reported to play a neuroprotective role against oxidative stress in astrocytes [14], oligodendroglia cells [21], retinal ganglion cells [22], and human lymphocytes [23]. The animal with DHA diet or transgenic *fat-1* mice rich in endogenous *n*-3 PUFA showed a better behavior performance [11,12,13,24]. H_2_O_2_ is a common oxidant to cause oxidative damage to cells and widely used in establishment of oxidative injury models [25]. Our present study reported that both exogenous and endogenous ω-3 PUFAs significantly protected NPCs against H_2_O_2_-induced oxidative injury, suggesting that ω-3 PUFAs might be an effective supplement for the prevention of neurodegenerative diseases which are associated with oxidative stress. DHA has been reported to scavenge the intracellular radical productions induced by hydrogen peroxide (H_2_O_2_), superoxide anion (O_2_^•−^), and hydroxyl radical (•OH) [22]. Many previous studies reported that DHA treatment could significantly reduce ROS production, which is a possible mechanism underlying DHA’s protective effects [14]. However, no significant differences in intracellular ROS levels were found between treatments in our study using 2′,7′-dichlorofluorescin diacetate (DCFH-DA) to measure intracellular ROS levels. A previous study reported that DHA at some certain concentrations showed no effects on the fluorescence change by use of ROS probe [26]. It could be a reason why we were unable to detect the differences in intracellular ROS levels between the DHA treatment and control group. Another possible reason may be the cell type used in our study. Our study investigated whether DHA could protect NPCs against oxidative injury. NPCs are capable of self-renewal and they grow and proliferate rapidly in the culture, which may make it difficult to accumulate ROS to sufficient levels for measurement inside cells. To detect ROS changes in NPCs may require more sensitive methods or probes. Regarding protective effects of DHA on attenuating oxidative stress/damage induced by H_2_O_2_, our study suggests that DHA exert its antioxidative effects possibly via initiating a translocation of Nrf2 from the cytoplasm to the nucleus and subsequently stimulating the expression of a battery of antioxidant and phase II detoxification molecules as a response to oxidative injury.

The nuclear factor erythroid 2-related factor 2 (Nrf2) is an emerging regulator of cellular resistance to oxidants [4,5,6]. Nrf2 is localized mainly in the cytoplasm bound to its specific repressor Keap1. Oxidative stress will cause the liberation of Nrf2 and allow it to translocate into the nucleus. Nrf2 will induce transcriptional upregulation of numerous antioxidant and phase II detoxification genes to provide efficient cytoprotection [27]. The activated Nrf2 has shown protective effects in animal models of many neurodegenerative disorders [28,29,30]. In the present investigation, we demonstrated that both exogenous and endogenous DHA enhanced Nrf2 translocation from the cytoplasm to the nucleus of cultured NPCs when exposed to the oxidative stress and subsequently stimulated the mRNA levels of Nrf2, GCLC, GCLM, NQO-1, and HO-1. These results confirm previous findings that treatment of DHA can induce antioxidant and detoxifying genes [31,32].

Although our study demonstrated a similar antioxidative effect between exogenous and endogenous DHA on cultured NPCs, a difference in mechanisms underlying protective effects of exogenous and endogenous DHA may exist. The fat-1 gene, which is absent in mammals, encodes omeg-3 polyunsaturated fatty acids (ω-3 PUFAs) that convert ω-6 to ω-3 PUFAs, leading to an elevated amount of ω-3 PUFAs such as DHA and higher ω-3 PUFAs/ω-6 PUFAs ratio in cells and tissues from fat-1 mice. PUFAs are essential components of membrane phospholipids and have a specific influence on membrane properties. Membranes enriched in ω-3 PUFAs show increased membrane fluidity [33] and can directly or indirectly affect the function of a number of membrane proteins such as receptors since receptors and their affinity to their respective hormones/growth factors/proteins depend on the fluidity of the cell membrane [34,35]. The increased membrane fluidity can be involved in antioxidative effects of endogenous DHA in addition to its direct action on initiating Nrf2 translocation from the cytoplasm to the nucleus, while exogenous DHA is considered to exert its antioxidative effects possibly via a direct action on initiating a translocation of Nrf2 from the cytoplasm to the nucleus and subsequently stimulating the expression of a battery of antioxidant and phase II detoxification molecules as a response to oxidative injury.

## 4. Experimental Section

### 4.1. Animals

We obtained *fat-1* breeders on a C57BL/6 background from Dr. Jing X. Kang (Harvard Medical School, Boston, MA, USA) and arose in the Laboratory Animal Center, University of Macau (Macau, China). Mice were housed in a temperature-controlled, 12:12 light/dark room and were allowed free access to water and food. The F1 progeny were obtained by mating C57BL/6 × C3H *fat-1* breeders with C57BL/6 WT mice. Generations of heterozygous fat-1 mice were then mated with WT littermates to obtain WT and heterozygous *fat-1* mice. All animals were treated in accordance with prevailing laws on animal experiments that were approved by the ethical committee of the University of Macau (Macau, China).

### 4.2. Genomic DNA Extractions and PCR Amplification

The *fat-1* C57BL6 mice (fat-1) and *fat-1* negative C57BL6 mice (WT) were identified by genotyping using PCR. Genomic DNA was prepared from 1 to 2 mm sections of tail tip using DNA Isolation Kit for Cells and Tissues (Roche, Mannheim, Germany). The DNA was used running polymerase chain reactions (PCR) using oligonucleotide primers that are specific for the transgene. Primer pair sets for the *fat-1* gene were constructed from Invitrogen (Carlsbad, CA, USA) and were as follows: Fat-1 forward: 5′-TGTTCATGCCTTCTT-CTTTTTCC-3′; reverse: 5′-GCGACCATACCTCAAACTTGGA-3′. PCR was carried out using rTaq (Takara, Otsu, Japa) with the following conditions: 95 °C 60 s (1 cycle); 95 °C 20 s, 58 °C 30 s, 72 °C 40 s (34 cycles). Amplified fragments were separated by 1.5% agarose gel electrophoresis.

### 4.3. Cell Isolation and Culture

Under sterile conditions, cerebral cortex from E13.5 *fat-1* mice and WT littermates were dissected out and prepared for NPCs culture following procedures described previously with minor modifications [25]. Briefly, the cortex was separated from surrounding tissues. After peeling off the meninges, the cortex was transferred into a 15 mL centrifuge tube containing culture medium (described below) and dissociated to a single-cell suspension by gentle mechanical trituration through a fire polished Pasteur pipette. The dissociated cells were filtered through a cell strainer (BD Falcon, Franklin Lakes, NJ, USA) and then cultured in T25 flask in suspension. The culture medium consisted of DMEM-F12, BSA (1 mg/mL), B27 (20 IU/mL), N2 (10 IU/mL), EGF (20 ng/mL), and bFGF (20 ng/mL). Cells were maintained in an incubator with a humidified atmosphere containing 5% CO_2_ at 37 °C. NPCs isolated from fat-1 mice and their WT littermates were confirmed by genomic DNA analyses and designated as NPCs^fat-1^ and NPCs^WT^ respectively. The medium was changed every two days. After five to six days, cells grew in neurospheres with the diameter of approximately 150 μm. Cells in the neurospheres were passaged at the ratio of 1:6 after initial plating. These subcultured cells were designated as “first passage” (P1). The third passage (P3) cells were used for all the following experiments. For differentiation studies, growth factors were removed from the culture medium and 1% fetal bovine serum (FBS, Gibco, Life Technologies Inc., Grand Island, NY, USA) was added. The cultures were allowed to differentiate for up to five days.

### 4.4. Exposure to H_2_O_2_ and Pretreatment with DHA

Dilutions of H_2_O_2_ (Sigma-Aldrich, St. Louis., MO, USA) were made fresh from a 30% stock solution into cell culture medium to the different terminal concentrations. The NPCs^WT^ were seeded at a density of 1 × 10^4^ cells per well into 96-well plates, then incubated in a humidified atmosphere of 95% air and 5% CO_2_ at 37 °C. A 200 μM H_2_O_2_ concentration in NPCs was determined to be optimal for this study (data not shown).

DHA (Sigma-Aldrich, St. Louis., MO, USA) was dissolved in 100% ethanol and kept at −20 °C in the dark as described in a previous study [24]. Immediately before use, the DHA stock solution was diluted in the bath solution and adjusted to the final concentrations needed. To examine the protective effects of DHA on H_2_O_2_-mediated apoptosis, NPCs^WT^ at a confluence of around 75% was pretreated with DHA (0, 0.1, 1, 10, and 50 μM) for 2 h and followed by oxidative injury induced by H_2_O_2_ treatment. NPCs^fat-1^ were exposed to H_2_O_2_ directly to investigate the protective effects of endogenous ω-3 PUFAs against oxidative injury. These cultures were then proceeded to cell viability analysis and TUNEL staining.

### 4.5. Analysis of Cell Viability and TUNEL

Cell viability was assessed using the WST-8 dye (Beyotime Inst Biotech, Haimen, China) according to the manufacturer’s instructions. After 10 μL WST-8 dye was add to each well, cells were incubated at 37 °C for 2 h and the absorbance was finally determined at 450 nm using a microplate reader (Molecular Devices, Sunnyvale, CA, USA). The results were expressed as relative cell viability (%). The apoptotic cell death of NPCs was estimated using TUNEL staining (Roche Applied Science, Indianapolis, IN, USA) according to the manufacturer's protocol. Cell cultures were counterstained with DAPI (5 μg/mL), which stained the nuclei of all cells.

### 4.6. Real-Time RT-PCR

Total RNA was extracted from NPCs^WT^, NPCs^fat-1^ and pretreated NPCs^WT^ with 10 μM DHA after exposed to 200 μM H_2_O_2_ for 6 h using TRIzol reagent (Invitrogen, Carlsbad, CA, USA) according to the manufacturer’s instructions. cDNAs were amplified and quantified in ABI Prism 7500 Sequence Detection System (Applied Biosystems, Foster City, CA, USA) using dye SYBR Green I (Takara, Otsu, Japan). The fold change in the levels of Nrf2, HO-1, GCLC, GCLM, and NQO-1 between the NPCs^WT^ and NPCs^fat-1^, normalized by the level of β-actin, was determined using the following equation: Fold change = 2^−∆(∆Ct)^, where ∆Ct = Ct(target) − Ct(β-actin) and ∆(∆Ct) = ∆Ct(treated) − ∆Ct(untreated). The primer sequences are listed in Table 1.

**Table 1:** Primers for real-time PCR assay.

Gene	Primer (5′-3′)
Nrf2	F: TTCTTTCAGCAGCATCCTCTCCAC
	R: ACAGCCTTCAATAGTCCCGTCCAG
NQO1	F: GCGAGAAGAGCCCTGATTGTACTG
	R: TCTCAAACCAGCCTTTCAGAATGG
HO-1	F: CAAGCCGAGAATGCTGAGTTCATG
	R: GCAAGGGATGATTTCCTGCCAG
GCLM	F: GCCACCAGATTTGACTGCCTTTG
	R: TGCTCTTCACGATGACCGAGTACC
GCLC	F: ACATCTACCACGCAGTCAAGGACC
	R: CTCAAGAACATCGCCTCCATTCAG
β-actin	F: TCGTGCGTGACATTAAGGAGAAG
	R: GTTGAAGGTAGTTTCGTGGATGC

### 4.7. Immunofluorescence

Immunocytochemistry was performed to characterize NPCs^WT^ and NPCs^fat-1^. Briefly, cells were fixed with 4% paraformaldehyde, blocked with 5% goat serum, and then incubated with primary antibodies overnight at 4 °C, including rabbit anti-nestin (1:1000, Millipore, Billerica, MA, USA), mouse anti-Rip (1:50, kindly gift from Dr. XM Xu, University of Louisville, Louisville, KY, USA), rabbit anti-GFAP (1:1000, Sigma-Aldrich, St. Louis., MO, USA), mouse anti-Tuj1 (1:1000, Sigma-Aldrich, St. Louis., MO, USA). The cells were then rinsed three times with PBS and incubated for 30 min with species-specific secondary antibody conjugated to the fluorescent labels Alexa 568 or 488 (1:400, Invitrogen, Carlsbad, CA, USA). Cell cultures were counterstained with DAPI (5 μg/mL) to stained the nuclei of all cells. Finally, the cells were visualized under a fluorescent laser microscope (IX73, Olympus Corp., Tokyo, Japan).

### 4.8. Western Blotting Analysis

Cells were washed twice with ice-cold PBS and lysed using a Nuclear and Cytoplasmic Protein Extraction Kit (Beyotime, Haimen, China) according to the protocol described by the manufacturer. The protein concentrations were determined using Bradford method. The protein extracts were separated in sodium dodecylsulfate polyacrylamide gel electrophoresis (SDS-PAGE) gel and then transferred to a poly-vinylidene difluoride (PVDF) membrane. They were then incubated overnight at 4 °C with primary monoclonal antibodies against Nrf2 (1:1000; R & D Systems, Minneapolis, MN, USA). Histone H3 (1:1000; Cell Signaling Technology, Beverly, MA, USA) and β-actin (1:1000; Cell Signaling Technology, Beverly, MA, USA) used as controls were detected in the nuclear fraction and cytosolic fraction, respectively. The blots were washed thoroughly in TBST buffer and incubated for 1 h with appropriate HRP-linked secondary antibodies (1:1000; Cell Signaling Technology, Beverly, MA, USA). Immunoreactive proteins were visualized with the ECL Western blotting detection reagent (Amersham Biosciences, GE Healthcare, Piscataway, NJ, USA). Relative band intensities were determined by Quality-one 1-D analysis software (Bio-Rad, Hercules, CA, USA).

### 4.9. Statistical Analysis

The results were expressed as the mean ± S.D. of triplicate measurements representative of three independent experiments. The one-way analysis of variance and Tukey test were used for the multiple comparisons. Statistical significance was defined as *P* < 0.05.

## 5. Conclusions

Both exogenous and endogenous DHA showed protective effects on NPCs against oxidative injury possibly via Nrf-ARE pathway, suggesting that DHA might be an effective supplement for the prevention of neurodegenerative diseases which are associated with oxidative stress.
